# Less is more: Optimizing antimicrobial prophylaxis in urological surgery

**DOI:** 10.1017/ash.2025.248

**Published:** 2025-09-24

**Authors:** Peter Oakes, Amy Mathers, Lindsay Donohue, Stacy Park, Heather Cox, Jacqueline Zillioux, Nicolas Ortiz, Alexander Henry

**Affiliations:** 1 Infectious Disease Fellow; 2 University of Virginia; 3 UVA Health; 4 University of Virginia Health System; 5 University of Virginia Health; 6 University of Virginia; 7 University of Virginia

## Abstract

**Background:** The selection of agent for antimicrobial prophylaxis for urological procedures is guided by the results of urine cultures taken prior to the day of surgery, which can lead to variability. National society guidance recommends single-dose antimicrobial prophylaxis immediately before urologic surgery; however, significant heterogeneity remains among practicing urologists with regards to pre-treating (or post-treating) bacteriuria identified on preoperative urine culture as well as choice of antimicrobial administered. As part of an institutional quality improvement initiative, we endeavored to optimize antimicrobial selection and duration of therapy through the use of a dedicated preoperative urine culture paired with recommendations. **Method:** This was a single-center, prospective study of urological surgeries. A dedicated preoperative urine culture was created in partnership with our institution’s microbiology lab and antimicrobial stewardship program intended solely for the selection of preoperative prophylaxis. Antibiotic stewardship program members reviewed these urine cultures and provided recommendations to urologic surgeons. Primary outcome was postoperative infectious complication within 90 days, with sub-analyses performed for stone and prosthetic cases, which carry higher infectious complication risks. **Result:** The preoperative urine culture was ordered prior to 381 urology cases from 9/27/23-4/15/24. There were 41 (10.8%) infectious postoperative complications. 64/381 (16.8%) patients received pretreatment for asymptomatic bacteriuria at the surgeon’s discretion, deviating from protocol recommendations for single-dose prophylaxis. Similarly, 44/381 (11.5%) patients received postoperative antimicrobials off-protocol per surgeon discretion. There was no statistically significant difference in infectious postoperative complication rates among patients who received pretreatment (15.6%, n=10/64) versus those who did not (9.8%, n=31/317 [p=0.18]), nor in those who received postoperative antimicrobials (13.6% (n=6/44) versus not (10.4%, n=35/337 [p=0.44]). Subgroup analyses of patients with nephrolithiasis or prosthetic material showed no benefit with supplemental antimicrobials. There were 294 total days of therapy in cases with guidance-based prophylaxis (n=294), and 611 days for pre- and/or post-treated cases (n=87), representing an excess of 524 days of antimicrobial therapy. **Conclusion:** We implemented a specific antimicrobial stewardship initiative linking a urine culture ordering process to succinct evidence-based advice. Deviation from advice did not result in improved outcomes but did result in excess antimicrobial days. Subgroup analysis also suggested single-dose prophylaxis is appropriate for patients considered higher risk for infectious complications. These findings support the recommendation from the American Urological Association that a single dose of antimicrobial prophylaxis is sufficient for the majority of urologic cases and demonstrate a multidisciplinary approach to ability to safely implement such practice.

